# Symptom co-fluctuations with suicidal ideation over time: a dynamic time warp analysis

**DOI:** 10.1136/bmjment-2026-302563

**Published:** 2026-07-23

**Authors:** A J C van der Slot, C Boonmann, M Eikelenboom, M W M Gijzen, A A L Kok, D De Beurs, B W J H Penninx, E J Giltay

**Affiliations:** 1 Department of Psychiatry, Leiden University Medical Center, Leiden, Zuid-Holland, Netherlands; 2 Department of Child and Adolescent Psychiatry, Curium-LUMC, Leiden, Zuid-Holland, Netherlands; 3 Psychiatric University Hospitals Basel (UPK), Basel, Switzerland; 4 Department of Psychiatry, Amsterdam UMC Location VUmc, Amsterdam, Netherlands; 5 Department of Pedagogy, University of Groningen, Groningen, Netherlands; 6 Department of Sociology, Vrije Universiteit Amsterdam, Amsterdam, Netherlands; 7 Department of Clinical Psychology, University of Amsterdam, Amsterdam, Netherlands; 8 Department of Public Health & Primary Care, Health Campus The Hague, Leiden University Medical Center, The Hague, Netherlands

**Keywords:** Anxiety Disorders, Depressive Disorder, Psychometrics

## Abstract

**Background:**

Suicidal ideation (SI) is a major global concern, yet its dynamic interplay with other symptoms remains poorly understood.

**Objective:**

To identify symptoms that co-fluctuate with or temporally precede SI to better understand symptom dynamics.

**Methods:**

Longitudinal data from three Dutch psychiatric cohorts with lifetime internalising disorders (16 waves from April 2020 until February 2022) were collected during the COVID-19 pandemic. We analysed depressive, happiness, anxiety, loneliness, worry symptoms and COVID-19-specific items only in those participants with SI fluctuations. Dynamic time warping (DTW) quantified within-person similarity between symptom trajectories and SI and results were aggregated at the group level.

**Findings:**

307 participants (mean age 44.8 years; 61.6% female) showed increasing SI over time (p<0.001). In the undirected analysis, SI aligned with four depressive symptoms (ie, sad mood, low self-esteem, low interest and reduced happiness), two anxiety-related symptoms (ie, fear of losing control, faintness) and overwhelming worrying, whereas the interpersonal symptoms feeling abandoned and feeling lonely did not survive false discovery rate (FDR) corrections. In directed analysis, while sad mood, hypersomnia, numbness and worrying about projects initially demonstrated temporal precedence, no directed lead-lag relationships survived FDR correction.

**Conclusion:**

SI is embedded in a broad symptom network beyond depression. These results underscore the value of time-sensitive, idiographic monitoring using tools like DTW to capture the person-specific temporal pathways through which SI emerges and intensifies.

**Clinical implications:**

This study suggests a core group of affective, cognitive and distress-related symptoms that could serve as informative signals for evaluating changes in SI and may represent actionable targets for intervention.

**WHAT IS ALREADY KNOWN ON THIS TOPIC?**

**WHAT THIS STUDY ADDS?**

- This study uses dynamic time warping to map within-person trajectories and identifies a set of affective, cognitive and panic/distress related symptoms that co-fluctuate with SI over weeks and months.

**HOW THIS STUDY MIGHT AFFECT RESEARCH, PRACTICE OR POLICY?**

- The results support a shift towards network-based models in suicidology, emphasising the need for time-sensitive monitoring to capture the complex and dynamic nature of suicidality.

## Introduction

 Suicidal ideation (SI) is a pressing global concern, with lifetime prevalence reaching 22.7%.^1^ Although linked to depression, this disorder’s heterogeneity limits its predictive power,^2^ underscoring the need to examine symptom-level dynamics rather than broad diagnostic categories.^3^ Evidence shows SI can arise independently of depressive severity, suggesting symptom dynamics beyond depression alone must be studied.^4^ Other affective and interpersonal states, such as anxiety and loneliness, are also recognised as proximal risk factors.^5^ Theoretical models, like the integrated motivational-volitional model,^6^ support this by conceptualising SI as emerging from dynamic interactions between factors like entrapment, fluctuating affect and interpersonal stress.^7^ However, it remains largely unclear how SI fluctuates over time and which symptoms co-fluctuate with its course, limiting timely detection and intervention.^8^ The COVID-19 pandemic constituted a period of substantial environmental instability, marked by elevated distress, loneliness and disruptions in daily life.^9^ This turbulent context provided a naturalistic setting with heightened variability in affective, cognitive and social experiences, offering an opportunity to examine how SI dynamically co-varies with these symptoms and context-related stress responses over time. Notably, empirical findings during the pandemic were mixed: some studies observed increases in SI,^9^ whereas others reported relative stability despite rising depression and anxiety.^10^ These divergent patterns underscore that SI does not simply track general distress and highlight the need for approaches that capture its dynamic within-person interplay with co-occurring symptoms and stressors. Additionally, such dynamics may differ across gender and age groups. Gender-specific patterns in risk expression suggest that different symptoms may drive the course of SI. In a cross-sectional network study in younger women, SI was closely linked to hopelessness and loneliness,^11^ whereas in men, low self-worth and perceived loss of control were more central.^12^

In this study, we applied dynamic time warping (DTW), a non-linear alignment algorithm that detects temporal patterns in intra-individual symptom trajectories despite irregular sampling and varying sequence lengths.^13^ DTW is particularly suited for mental health research, where the assumption that group-level patterns mirror individual processes (ergodicity) is frequently violated.^14^ DTW can also assess temporal directionality, providing preliminary insight into which symptoms may precede or follow changes in SI.^13^ Recent work in psychopathology has emphasised that mental disorders emerge from dynamic symptom interactions rather than static traits.^14 15^ Network models provide a useful framework by conceptualising symptoms as interconnected nodes with potential temporal directionality.^15^ In this study, we use DTW to calculate the temporal distances between symptom trajectories. The resulting distance matrix then serves as the basis for a network analysis. This combination allows for the identification of symptom clusters based on shared, non-linear temporal patterns, moving beyond the static correlations of traditional models. Early psychiatric applications of DTW have demonstrated its value for modelling personalised dynamics, yet only two studies have examined SI at the individual level.^7 16^ Building on ecological momentary assessment (EMA) research showing that SI and its proximal risk factors fluctuate markedly within individuals over hours to days,^17^ these studies applied DTW to EMA data, identifying key roles for entrapment and rumination and showing that low inner peace preceded suicidality. However, their small sample sizes (n=11 and n=28) limited generalisability beyond short-term within-day fluctuations. Therefore, in this study we investigate which affective, cognitive and interpersonal symptoms co-fluctuate with SI across weeks and months, whether certain symptoms temporally precede changes in SI and whether these alignments differ by sex and age. Mapping these dynamics can deepen our understanding of the symptom dynamics underlying suicidality and may provide novel approaches for uncovering the dynamic processes through which suicidality emerges and intensifies.

## Methods

### Study population and procedure

Data were integrated from three Dutch longitudinal psychiatric cohorts: the Netherlands Study of Depression and Anxiety (NESDA; n=2329; ages 18–65 years), recruited from primary and specialised care with current or lifetime depressive and/or anxiety disorders,^18^ the Netherlands Study of Depression in Older Persons (NESDO; n=510; ages 60–93 years), focusing on late-life depression excluding dementia^19^ and the Netherlands Obsessive Compulsive Disorder Association Study (NOCDA; n=419; ages 18–65 years) with lifetime obsessive-compulsive disorder.^20^ Diagnoses were established using the Composite International Diagnostic Interview (CIDI) or Structured Clinical Interview for the Diagnostic and Statistical Manual of Mental Disorders DSM-IV (SCID-I). Participants with primary psychotic or severe neurological disorders were excluded. These cohorts employ harmonised diagnostic procedures and data collection protocols; detailed descriptions of the study designs, recruitment procedures and assessment batteries are provided elsewhere.^18–20^

### Participants

During the COVID-19 pandemic (April 2020–February 2022), 2748 participants were invited for up to 16 waves of online questionnaires. Of the 1125 individuals who completed at least one assessment, we included those with:

1. Complete data for at least four waves on all relevant items.
2. At least one within-person fluctuation in SI (≥ point change on Quick Inventory of Depressive Symptomatology Self Report (QIDS)-SR16 item 12).

The final analytical sample (n=307) consisted of participants from NESDA (n=252), NESDO (n=11) and NOCDA (n=44). Across the 16 waves, the median interval was 35 days (IQR: 26–42 days; range: 14–220 days).

**Figure 1:**
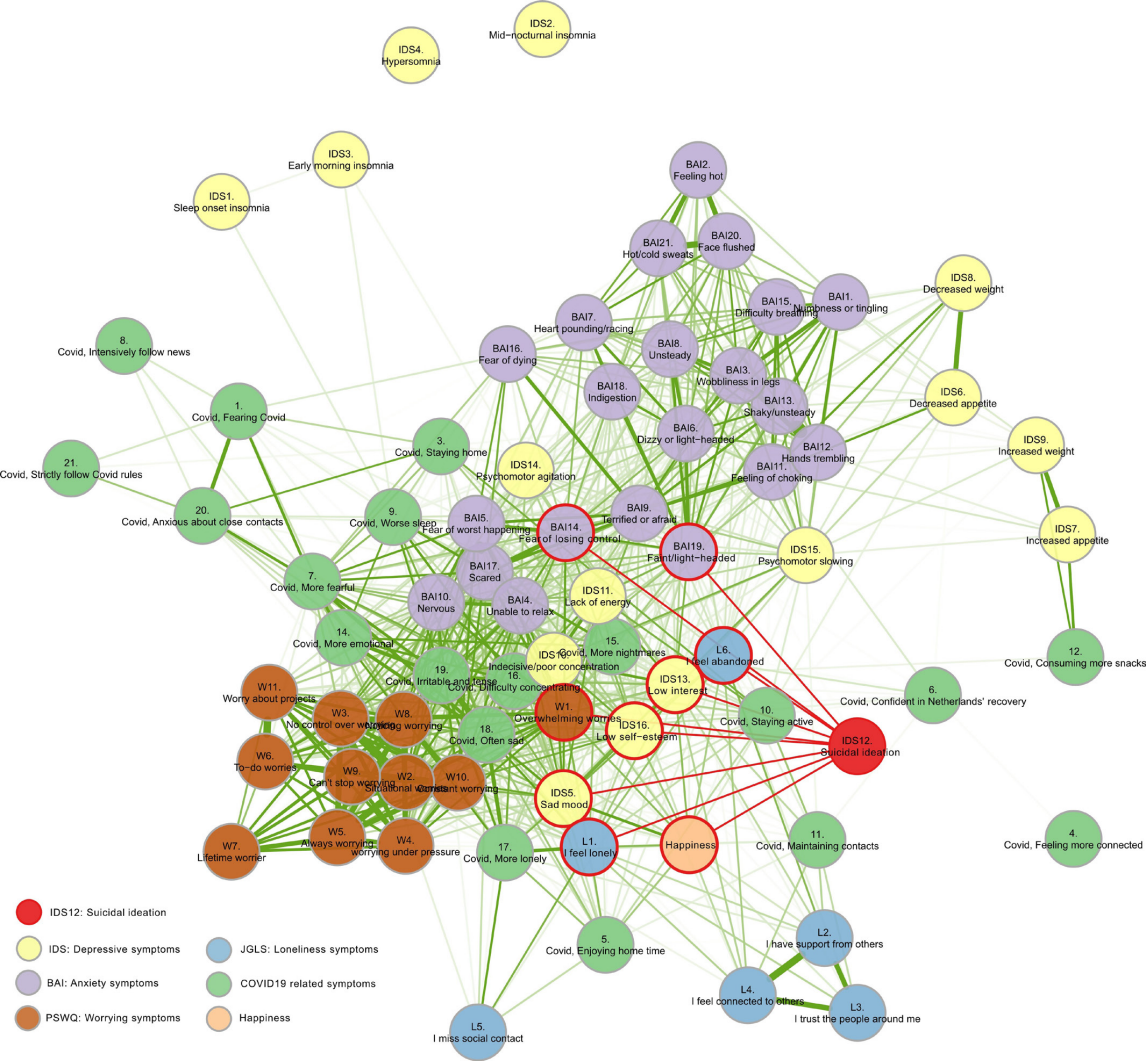
Undirected symptom network across all domains. Nodes represent individual items. Edges reflect significant partial correlations between items, with edge thickness proportional to strength. Node colour separates symptom domains. The network is undirected and based on pooled data across all 307 participants. BAI, Beck Anxiety Inventory; IDS, Inventory of Depressive Symptomatology; JGLS, de Jong-Gierveld Loneliness Scale; PSWQ, Penn State Worry Questionnaire.

While included and excluded participants did not differ in age, sex or educational level, the included sample showed significantly higher comorbidity (see online supplemental table 1). For the age-stratified visualisations and analyses (eg, figure 1), the sample was divided using a 60 year threshold. This cut-off was chosen for both structural and substantive reasons. First, it aligned with the recruitment criteria of the contributing cohorts, as NESDO specifically included adults aged 60 years and older, while NESDA covered the younger age range. Second, this threshold is consistent with United Nations frameworks, which commonly define older persons as individuals aged 60 years or over. Online supplemental figure 1 shows the inclusion/exclusion flowchart. The study protocol was approved by the institutional review board of the Vrije Universiteit Medical Center, Amsterdam (ref. 2020.166), and participants provided informed consent digitally.

### Instruments

Mental health outcomes during both pre-pandemic and COVID-19 measurement waves included:

SI was assessed with item 12 of the 16-item QIDS-SR16. Response options range from 0 = ‘I do not think of suicide or death’, 1 = ‘I feel that life is empty or wonder if it is worth living’, 2 = ‘I think of suicide or death several times a week for several minutes’ to 3 = ‘I think of suicide or death several times a day in depth or have made specific plans or have actually attempted suicide’. This item served as the primary dependent variable.

Depressive symptoms were assessed with the remaining 15 items of the QIDS-SR16, (α ≈ 0.81–0.86).^18^

Anxiety symptoms were assessed with the 21-item Beck Anxiety Inventory (BAI; α ≈ 0.92–0.94).^18^

Loneliness symptoms were measured with the six-item De Jong Gierveld Loneliness Scale (α ≈ 0.77–0.79).^18^

Worry was assessed using the 11-item Penn State Worry Questionnaire (PSWQ-11; α ≈ 0.93).^18^

Happiness was evaluated with the single-item Self-Rating of Happiness scale (range 1–7), reverse-coded for analysis so higher scores indicate lower happiness.^18^

COVID-19 stressors were captured via 21 items covering emotional impact, coping and adherence to measures (on a five-point Likert scale).^18^ The digital questionnaires were constructed using online survey software (Survalyzer). Although exact completion times were not recorded, the whole assessment that comprised the above mentioned 74 standardised multiple-choice and Likert-scale items took approximately 15–20 min to complete.

### Covariates

Baseline sociodemographic covariates included age, sex and years of education using in the mixed models to yield the two forest plots. Clinical variables comprised lifetime diagnoses of major psychiatric disorders (major depressive disorder (MDD), general anxiety disorder (GAD), panic disorder and obsessive compulsive disorder) established via the CIDI or SCID-I.^18–20^

Two indices of psychiatric burden were computed: (1) the total number of lifetime disorders, reflecting diagnostic comorbidity, and (2) chronicity, categorised as no current disorder, ≤50% of preceding waves with a current disorder or >50% of waves with a current disorder.

### Statistical analysis

Baseline characteristics were summarised using means (SD), medians (IQR) or counts (%) as appropriate, stratified by age and sex. Temporal trends in SI over the 22-month pandemic period were visualised alongside national COVID-19 mortality data and tested using a linear mixed-effects model with time as a fixed effect and participant as a random intercept.

Prior to analysis, all items were standardised (z-scores) and harmonised so that higher scores consistently represented greater severity. To ensure robustness against outliers and irregular sampling, scores were winsorised (capped at ±3). To optimise the DTW alignment, five intermediate points were linearly interpolated between each pair of consecutive assessments. This step was taken specifically to increase the temporal resolution of the trajectories, thereby reducing artefacts caused by mismatched start and endpoints, rather than to impute missing primary data.

To examine the temporal alignment between SI and other variables, we first applied an undirected DTW to participant-level panel data. DTW is a non-linear alignment technique that quantifies the similarity between two time-series by identifying the optimal match between their trajectories, even when the timing of fluctuations differs.^21^ This involves constructing a cost matrix where each cell represents the distance between two points in time. The ‘optimal match’ is then found by tracing the path through this matrix that minimises the total cumulative distance between the symptom levels, effectively aligning asynchronous peaks. This approach captures parallel within-person changes regardless of irregular spacing between assessments. A time-window constraint (Sakoe-Chiba band) of 1 allowed each observation to align with points one wave earlier or later, emphasising co-fluctuation occurring at or near the same assessment.^13^ The method is well suited for the relatively short and unevenly spaced series collected here (16 waves per participant over 22 months). For each participant, pairwise DTW distances were computed across all 74 items. This yielded 307 individual (74 by 74) DTW distance matrices. Throughout, we use the term ‘symptoms’ to refer to all included items, acknowledging that the COVID-19 variables represent contextual stressors and behaviours. At the group level, each symptom pair’s DTW distance was compared against the average distance of all other symptom pairs. P values tested whether a pair’s distance was significantly smaller than the empirical mean, corresponding to the null hypothesis of independent fluctuation. Only symptom pairs with significantly smaller distances (p<0.05) were retained. This undirected network reflects unadjusted associations and served as the descriptive basis for the adjusted mixed-effects analyses presented in the forest plots and online supplemental figures. Linear mixed-effects models estimated the relative strength of alignment between SI and each symptom. For the undirected contemporaneous analysis, significance testing was strictly one-sided, evaluating which individual symptom exhibited a DTW distance that was significantly smaller than the average distance of the remaining 72 symptoms. To account for alpha-error inflation resulting from multiple comparisons across these 73 concurrent comparisons, we applied Benjamini-Hochberg (BH) false discovery rate (FDR) corrections (α<0.05).

To further explore temporality, we applied a directed DTW analysis using an asymmetric window to estimate lead-lag relationships.^13^ Rather than computing a single symmetric index from the outset, directionality was operationalised through the asymmetric Sakoe-Chiba alignment window that constrained the trajectories to look only one time point ahead in the hypothesised direction. Crucially, to avoid directional artefacts, the local cost function itself was strictly symmetric, using the symmetric two step pattern. For each participant and each symptom-SI pair, we computed two independent alignment costs: one where the symptom trajectory was constrained to precede SI and one reverse ordering. A net relative directed distance index was then calculated for each pair by computing the relative difference between these two costs. By dividing this difference by the sum of the two distances, the index could range from −1 (ie, scores are exactly following in time) to 1 (scores are exactly preceding in time). A step-by-step visual and mathematical walkthrough of this matrix alignment and index calculation is provided in online supplemental figure 2. At the group level, these individual directed indices were subjected to two-sided one-sample t-tests against a mean baseline of zero (p<0.05). In this context, a value of zero denotes perfect temporal synchrony, where neither trajectory systematically precedes the other. A statistically significant positive deviation from zero identifies a symptom as a systematic precursor (ie temporal lead), summarised via out-strength centrality, whereas a negative deviation reflects a lagging response, summarised via in-strength centrality. To also control for multiple comparisons across the 73 directional comparisons, a separate BH FDR correction was applied (α<0.05). Within the manuscript and figures, we explicitly denote which contemporaneous alignments and directed temporal precursors successfully survived their respective multitesting adjustments.

All statistical analyses were performed using the packages ‘dtw’ (V.1.231), ‘parallelDist’ (V.0.2.6), ‘forestplot’ (V.3.1.3) and ‘qgraph’ (V.1.9.8) of the statistical programme R Studio V.4.3.2; R Foundation for Statistical Computing, Vienna, Austria, 2016 (https://www.R-project.org/).

## Results

### Patient characteristics

The 307 included participants completed an average of 10.7 assessments (SD=3.71, range=4–16) and had a mean age of 44.8 years (SD=11.2), with 61.6% being female and 49.5% having completed a high level of education. Lifetime diagnoses were common in this group, with all having a history of a mental disorder, and high rates of lifetime MDD (65.8%), GAD (31.4%) and panic disorder (32.7%). Regarding functional outcomes during the pandemic, the sample had significantly higher scores on average depression (QIDS), anxiety (BAI) and loneliness (PWSQ) scores, compared with excluded participants (all p<0.001). Included participants also had more lifetime disorders and had a higher chronicity of disorders compared with excluded individuals (both p<0.001), as assessed through CIDI/SCID-based diagnostic interviews. Table 1 shows the sociodemographic and clinical characteristics of the four demographic subgroups defined by age and sex (n=307).

**Table 1:** Comparison of baseline characteristics in the four demographic subgroups of the sample (307 participants)

	Current study*				P value†
	Men <60 years (n=56)	Women <60 years (n=104)	Men ≥60 years (n=62)	Women ≥60 years (n=85)	
Sociodemographics					
Age	50.4 (7.9)	48.1 (8.1)	69.6 (5.6)	68.4 (4.5)	
High level of education	35 (62.5%)	66 (63.5%)	33 (53.2%)	55 (64.7%)	0.50
Comorbidity					
Lifetime MDD diagnosis	115 (65.3%)	284 (69.3%)	136 (60.4%)	205 (65.3%)	0.16
Lifetime GAD diagnosis	54 (30.7%)	149 (36.3%)	57 (25.3%)	93 (29.6%)	0.029
Lifetime PD diagnosis	53 (30.1%)	143 (34.9%)	63 (28.0%)	109 (34.7%)	0.24
Lifetime OCD diagnosis	31 (17.6%)	47 (11.5%)	8 (3.6%)	9 (2.9%)	**<**0.001
Number of lifetime disorders	3.50 (1.51)	3.31 (1.32)	3.26 (1.40)	3.48 (1.37)	0.65
Chronicity					0.73
Lifetime disorders but 0% chronicity	10 (17.9%)	16 (15.4%)	14 (22.6%)	16 (18.8%)	
1–50% chronicity	14 (25.0%)	37 (35.6%)	16 (25.8%)	27 (31.8%)	
51–100% chronicity	23 (57.1%)	51 (49%)	32 (51.6%)	42 (49.4%)	
Clinical characteristics					
Depressive symptoms (QIDS)	10.2 (4.8)	10.2 (4.7)	9.1 (4.9)	9.4 (4.1)	0.44
Anxiety symptoms (BAI)	14.0 (10.6)	15.7 (11.3)	13.1 (10.8)	12.5 (9.2)	0.29
Worrying symptoms (PSWQ)	37.7 (9.2)	39.5 (10.0)	32.1 (11.9)	33.5 (9.4)	**<**0.001
Loneliness symptoms (JGLS)	3.8 (1.6)	3.7 (1.9)	3.9 (1.8)	3.9 (1.8)	0.86

* Participants without a lifetime psychiatric diagnosis and those without any change in suicidal ideation (IDS item 12) during the pandemic were excluded from the analytical sample. Data are presented as mean (SD) for continuous variables and n (%) for categorical variables.

† P values were estimated using χ2 tests for categorical variables, t-tests for normally distributed continuous variables and Kruskal-Wallis tests for non-normally distributed continuous variables. Clinical characteristics reflect baseline scores prior to the COVID-19 assessment period. Comorbidity was assessed with CIDI diagnostic interviews based on Diagnostic and Statistical Manual of Mental Disorders (DSM-IV) criteria. Ranges: QIDS (0–27), BAI (0–63), PSWQ (16–90) and JGLS (0–6).

BAI, Beck Anxiety Inventory; CIDI, Composite International Diagnostic Interview; GAD, general anxiety disorder; JGLS, de Jong-Gierveld Loneliness Scale; MDD, major depressive disorder; OCD, obsessive compulsive disorder; PD, panic disorder; PSQW, Penn State Worry Questionnaire; QIDS, Quick Inventory of Depressive Symptomatology.

### Sample trends in SI

Online supplemental figure 3 displays temporal trends in SI across the sample (n=307). Mean SI scores increased significantly over time (p<0.001, top panel), while the bottom panel shows weekly COVID-19 mortality in the Netherlands annotated with major public health measures (eg, lockdowns, curfews). Only a slight correspondence was observed between temporary increases in mean SI and peaks in COVID-19 deaths or restrictions.

### Undirected symptom network structure

Figure 1 presents the undirected DTW symptom network, retaining only edges between symptom pairs with significantly smaller DTW distances than expected. The network shows moderate clustering of symptoms within their original scales (eg, IDS, BAI, PSWQ), but also notable cross-domain connections, suggesting interrelations across symptom domains. SI (IDS12), highlighted in red, was positioned at the periphery of the network and maintained connections with key symptoms from multiple domains. Significant connections were observed with depressive symptoms, including low interest (IDS13), low self-esteem (IDS16) and sad mood (IDS5), as well as with the loneliness symptoms ‘feeling abandoned’ (L6) and ‘feeling lonely’ (L5). Additional connections were found with cognitive-affective anxiety symptoms ‘fear of losing control’ (BAI14), ‘faintness’ (BAI19) and reduced happiness. In contrast, COVID-specific symptoms clustered peripherally, with no direct connections to SI.

### Symptom-specific alignments with SI (forest plot results)

**Figure 2:**
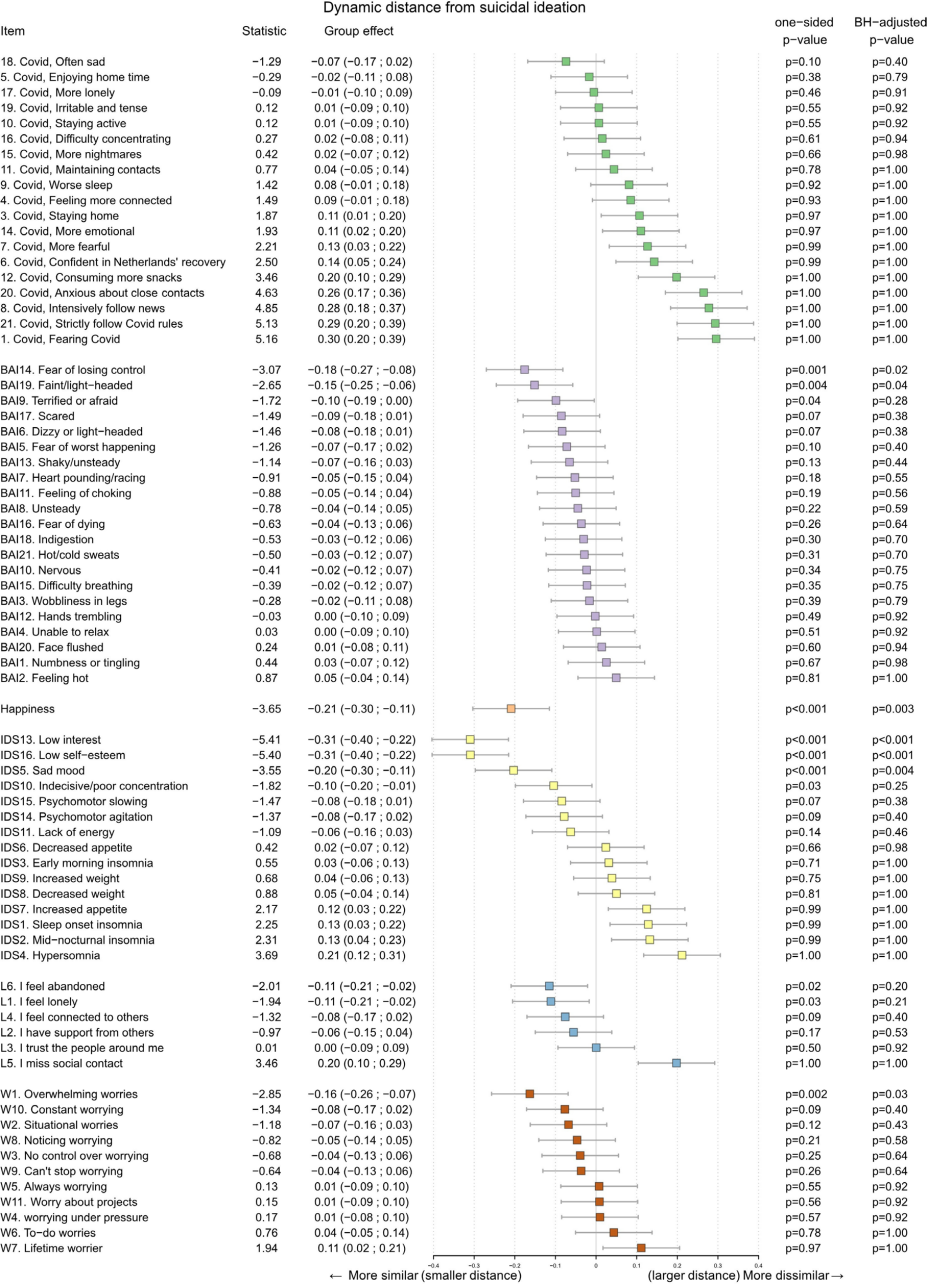
Undirected dynamic distances from suicidal ideation across all domains. This figure summarises the relative dynamic time warping distances between individual symptoms and SI across six operationalised domains: COVID-19-related stressors, anxiety (BAI), depression (IDS), interpersonal loneliness (JGLS), worry (PSWQ) and happiness. The zero point represents the average distance across all investigated items; negative group effects indicate a closer trajectory similarity (stronger contemporaneous alignment) with SI, whereas positive values indicate weaker alignment. Two significance thresholds are presented: (1) one-sided p values representing the initial unadjusted clinical alignments (p<0.05) and (2) Benjamini-Hochberg (BH)-adjusted p values representing the final significance levels after applying the stringent BH false discovery rate correction to control for multiple comparisons across all 73 comparisons. Error bars represent 95% CIs around the estimated dynamic distances. BAI, Beck Anxiety Inventory; IDS, Inventory of Depressive Symptomatology; JGLS, de Jong-Gierveld Loneliness Scale; PSWQ, Penn State Worry Questionnaire.

We examined DTW distances between SI and each of the 73 symptoms, adjusting for age, sex and educational level, to identify which symptoms co-fluctuated most closely with SI. Results are displayed in a forest plot (see figure 2), showing both the unadjusted and BH adjusted significance levels to control for multiple comparisons. Smaller DTW distances indicate stronger temporal alignment with SI, and thus indicate symptoms that co-vary more closely with SI. In the primary unadjusted analysis, several affective symptoms (ie,‘sad mood’, ‘low self-esteem’, ‘low interest’ and ‘indecisive/poor concentration’) together with the separate happiness symptom showed significant alignment with SI. Three anxiety symptoms (‘Terrified/afraid’, ‘fear of losing control’ and ‘feeling faint or light-headed’) and the cognitive symptom ‘overwhelming worrying’ were also significantly aligned with SI. In addition, the interpersonal symptoms ‘I feel lonely’ and ‘I feel abandoned’ co-fluctuated significantly with SI.

After applying the BH FDR correction across all 73 individual comparisons, a set of affective, cognitive and panic/distress-related symptoms survived the multitesting adjustment: four affective symptoms (sad mood, low self-esteem, low interest and reduced happiness), the cognitive symptom overwhelming worrying and the panic related symptoms fear of losing control and faintness. By contrast, none of the COVID-specific and somatic symptoms showed significant alignment.

Post-hoc subgroup analyses by sex and age indicated no significant effect modification of these associations, suggesting consistency across demographic groups (online supplemental figures 4-8 for detailed results).

Sensitivity analyses were conducted by stratifying the sample based on baseline depression (IDS <26 vs IDS ≥26) and anxiety severity (BAI <33 vs BAI ≥33). These analyses showed that in both lower-severity subgroups, affective symptoms co-fluctuated significantly stronger with SI than in the high-burden groups (see online supplemental figures 9, 10). Additionally, a sensitivity analysis stratifying participants by the within-person SD of the SI item (low variability, n=155, vs high variability, n=152) demonstrated largely similar symptom-SI alignment patterns between the two variability subgroups (see online supplemental figure 11).

Finally, to ensure that the pooled results were not driven by cohort-specific characteristics, sensitivity analyses were conducted by restricting the sample to the largest cohort (NESDA-only, n=241). The results were highly consistent with the primary analyses, showing stable rank-orderings for both contemporaneous symptom-SI alignments and lead-lag patterns, despite slightly wider CIs due to the reduced sample size (see online supplemental figures 12-14).

### Directed temporal associations with SI

**Figure 3:**
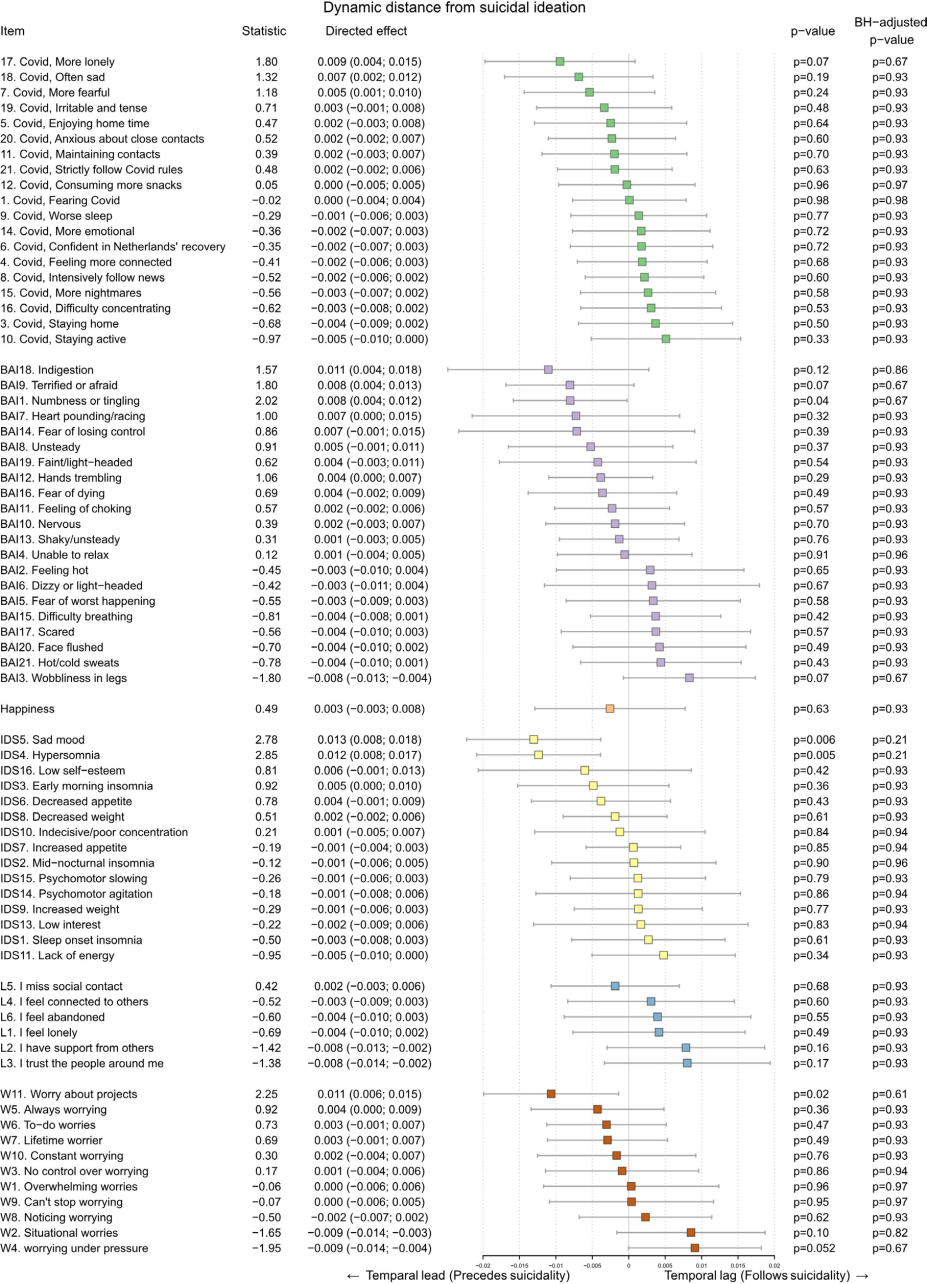
Directed dynamic distances from suicidal ideation across all domains. This figure displays the directed dynamic time warping lead-lag effects for all individual symptoms in relation to SI. Negative values indicate a lagging effect where the symptom tends to follow fluctuations in SI, whereas positive values indicate a leading effect where changes in the symptom chronologically precede changes in SI. Items are grouped by domain: anxiety (BAI), depression (IDS), interpersonal loneliness (JGLS), worry (PSWQ), happiness and COVID-19 stress-related and behaviour-related symptoms. Two significance thresholds are presented: (1) one-sided p values representing the initial unadjusted clinical alignments (p<0.05) and (2) Benjamini-Hochberg (BH)-adjusted p values representing the final significance levels after applying the stringent BH false discovery rate correction to control for multiple comparisons across all 73 comparisons. Error bars represent 95% CIs around the estimated directed effects. BAI, Beck Anxiety Inventory; IDS, Inventory of Depressive Symptomatology; JGLS, de Jong-Gierveld Loneliness Scale; PSWQ, Penn State Worry Questionnaire.

Figure 3 shows the forest plot displaying the directed DTW results, indicating which symptoms tended to statistically significantly precede or follow fluctuations in SI at the group level. Positive directed values reflect temporal lead, symptoms that typically rise before increases in SI, whereas negative values reflect temporal lags.

In the unadjusted analysis (p<0.05), four symptoms showed significant leading associations with SI: sad mood, hypersomnia, worrying about projects and numbness/tingling, suggesting that increases in these symptoms tended to precede subsequent increases in SI. No symptoms demonstrated significant lagging effects, suggesting that no specific symptom reliably followed changes in SI. After applying the BH FDR correction, directed comparisons to account for multiple comparisons, no directed temporal lead or lag relationships survived the multitesting adjustment (all adjusted p values ≥0.21)

## Discussion

This proof-of-principle, hypothesis-generating study explored whether DTW could identify clinically meaningful temporal patterns in longitudinal symptom data. It mapped the dynamic, within-person interplay between SI and a broad range of affective, cognitive, panic/distress and interpersonal symptoms using DTW. In the undirected DTW analysis, the study identified a set of symptoms co-fluctuating with SI, clustering around affective dysregulation (ie, sad mood, low self-esteem, low interest, reduced happiness), cognitive overload (ie, overwhelming worry) and panic/distress (ie, fear of losing control and faintness). After FDR corrections indecisiveness, feeling terrified and both interpersonal symptoms (ie, feeling lonely and feeling abandoned) were no longer statistically significant. None of the COVID-19-specific stressors and concerns showed a significant temporal alignment with SI, underscoring the greater role of internal psychological processes. In the directed analysis, changes in sad mood, hypersomnia, feeling numb and worrying about projects temporally preceded similar changes in SI, although these were no longer statistically significant after FDR corrections.

The alignment of SI with core affective symptoms such as sadness, low self-worth and low interest supports the view of suicidality as a fluctuating, state-like phenomenon shaped by proximal emotional experiences.^17^ The alignment with overwhelming worry, fear of losing control and faintness suggests that acute experiences of emotional and cognitive overwhelm or loss of agency could temporally align with SI, consistent with implicit cognition studies suggesting that some individuals perceive possible death as a means of regaining control.^22^ Furthermore, while the interpersonal symptoms feeling lonely and feeling abandoned lost significance after multiple comparisons control, their initial unadjusted alignments highlight a possible role of interpersonal distress. However, whether these relational dynamics act as stable correlates warrants replication in purpose-built intensive time-series studies. Although pandemic-related stressors have been linked to SI elsewhere,^9^ our analyses showed no temporal alignment of the COVID-19 symptoms with SI. This suggests their effects may be small, more distal or could be linked via symptoms more proximal to SI like sadness, panic or feelings of abandonment.^9^ Together, these results emphasise that SI is characterised by a dynamic interplay between affective, cognitive, distress-related and possibly interpersonal states, whereas external stressors appear to show indirect chronological relationships via these processes.

Sensitivity analyses further revealed that these symptom-SI co-fluctuations were significantly more pronounced in participants with lower baseline severity. Among individuals with less severe levels of depression and anxiety, SI appears to be more clearly associated with fluctuations in several anxiety and panic symptoms. In contrast, in high-burden subgroups, these associations were markedly less distinct, suggesting a form of ‘decoupling’ where SI becomes less synchronised with specific temporal fluctuations of other symptoms.^3 23^ Although the tighter alignment in lower-severity groups could theoretically reflect consistently low scores on both affective symptoms and SI, this is unlikely to be a purely statistical artefact, as we adjusted for the within-person SD of these scores at the individual level in our mixed model analyses. To further address this issue, we conducted an additional sensitivity analysis in which the 307 participants were stratified according to the within-person SD of the IDS SI item (item 12). Participants were divided into a low SI variability group (n=155) and a high SI variability group (n=152). The results showed relatively few differences between these two groups, suggesting that the observed severity-dependent pattern is unlikely to be driven solely by differences in SI variability (see online supplemental figure 11). The robustness of these findings, encompassing both the contemporaneous symptom-SI alignments and the temporal lead-lag patterns, is further supported by sensitivity analysis restricted to the largest single cohort (NESDA). Showing high consistency with the primary analyses, this suggests that the observed dynamics represent a stable phenomenon and are not unduly influenced by cohort-specific characteristics (case-mix).

### Implications

Methodologically, this study demonstrates both the utility and the clear boundaries of applying DTW to existing psychiatric datasets. On one hand, our findings show that DTW can accommodate irregular, non-stationary data typical of naturalistic cohorts.^21^ Even with lower-frequency assessments over weeks and months, DTW can recover meaningful symptom co-fluctuations, complementing EMA studies that capture within-hours/days SI dynamics.^17^ More broadly, these findings may point to the potential importance of idiographic, within-person approaches, as a hypothesis that warrants further investigation, particularly given that group-level associations may not fully generalise to individual processes.^14^ They support a shift toward models that conceptualise suicidality as functioning as dynamic symptom networks rather than fixed latent constructs.^8 15^ Integrating DTW with network modelling or digital phenotyping may further enhance risk prediction and contribute to real-time early warning systems.^15^

Clinically, our findings provide a more nuanced understanding of how suicidality is reinforced by dynamic symptom processes. The identified co-fluctuations suggest that affective dysregulation, intense worry, panic and potentially severe interpersonal distress can serve as informative signals when evaluating changes in suicide risk in high-risk populations. Recognising and discussing these co-occurring symptoms may also help reduce stigma and facilitate conversations about suicidality. For intervention, these symptoms represent actionable targets; for instance, increases in worry or perceived loss of control can inform safety planning and tailored therapeutic focus.^9^ Importantly, the lack of consistent leading effects after FDR correction across the full symptom set may partly reflect limited statistical power but may also reflect the clinical heterogeneity of suicidal processes. In addition, the study was not specifically designed to examine DTW-based temporal fluctuation patterns in relation to SI nor was it conducted in a group at high risk for SI. Rather than following a uniform trajectory, suicidality is likely characterised by multiple person-specific temporal sequences.^8^

Future digital tools may facilitate such monitoring. Purpose-built high-density designs such as EMA and passive sensing could enable tracking of symptom fluctuations in daily life. While traditional idiographic models often rely on vector autoregression to estimate predictive relations, DTW captures temporal synchrony in symptom trajectories without assuming fixed temporal lags. Together, these dynamic approaches illustrate how personalised symptom monitoring may contribute to more clinically meaningful suicide risk assessment.^7 17^

### Limitations and strengths

Several limitations should be noted. First, although the number of assessments was sufficient, their spacing was irregular ranging from biweekly to several months (median 35 days). Such variability complicates time-series analysis and may have obscured rapid short-term fluctuations in SI that may have occurred between waves. While DTW is less adversely affected by non-stationary and unevenly spaced data than parametric methods such as Multilevel Vector Autoregression,^21^ this variability poses a conceptual challenge. Some have argued that measurement intervals should align with the timeframe in which symptoms are theoretically expected to change.^23^ Second, SI was measured with a single ordinal QIDS-SR16 item (0–3), which provides a relatively coarse indicator; however, intensive longitudinal designs inevitably require a trade-off between the use of validated multi-item instruments and the feasibility and participant burden associated with frequent repeated assessments. Third, requiring at least one fluctuation in SI may have limited generalisability, particularly because individuals with a stable absence of SI were excluded. Fourth, participants were middle aged and older individuals with a Western-European ethnic background and lifetime affective disorder histories, limiting generalisability to younger and other ethnic groups of subjects. Fifth, requiring ≥4 assessments may have led to a selection bias toward more compliant participants. Sixth, directed effects at the group level were small, likely due to substantial between-person heterogeneity and measurement error of assessing individual symptoms through single items. Lastly, while DTW captures temporal synchrony between symptoms, it does not permit causal inference.

Despite these limitations, the study has several strengths. First, it applied DTW to model intra-individual symptom dynamics in a large harmonised cohort, providing a naturalistic test of SI trajectories during the COVID-19 pandemic. Second, the within-person modelling approach aligns with calls for idiographic, temporally sensitive models in psychiatry.^3^ Third, participants completed on average 10.7 assessments, offering a solid basis for panel data analyses using DTW.

## Conclusion

In conclusion, this study identified a consistent set of affective (sad mood, low self-esteem, low interest and reduced happiness) and cognitive-panic symptoms (overwhelming worries, fear of losing control and faintness) that co-fluctuate with SI across weeks to months, regardless of age or sex, whereas COVID-19-specific stressors played only a limited role. No directional lead-lag relationships remained statistically significant after FDR correction at the group level. Together, these findings underscore the value of within-person monitoring for understanding the dynamic mechanisms through which suicidality emerges while highlighting the need for future purpose-built high-frequency time-series studies to advance more personalised approaches to suicide risk detection and intervention.

## Supplementary material

10.1136/bmjment-2026-302563 online supplemental figure 1

## Data Availability

Data may be obtained from a third party and are not publicly available.
