# Supplementary material for: Symptom co-fluctuations with suicidal ideation over time: a dynamic time warp analysis
Source: BMJ Ment Health. 2026 Jul 23;29(1):e302563. doi: 10.1136/bmjment-2026-302563 (PMC13410913; doi:10.1136/bmjment-2026-302563)
Supplement: Supplementary file 2 [file bmjment-29-1-s001.docx]

Supplementary Materials

This appendix has been provided by the authors to give readers additional information about their work.

Supplement to: A.J.C. van der Slot, C. Boonmann, M. Eikelenboom, M.W.M. Gijzen, A.A.L. Kok, D. de Beurs, B.W.J.H. Penninx and E.J. Giltay. Symptom co-fluctuations with suicidal ideation over time: a dynamic time warp analysis.

**Supplementary materials**

Manuscript title: Within-person temporal alignment shows symptom co-fluctuations and early precursors of suicidal ideation. Authors: A.J.C. van der Slot, C. Boonmann, A.A.L. Kok, M.W.M. Gijzen, M. Eikelenboom, D. de Beurs, B.W.J.H. Penninx and E.J. Giltay									           

*Overview of supplementary content:* 						             	page

Supplementary Figure 1 	Flow chart of inclusion/exclusion process			    iii

Supplementary Table 1		Baseline characteristics of included vs. excluded participants	 iv-v

DTW sample script									                   vi

COVID-19 questionnaire		COVID-19-specific exposures and responses (21 items)		   vii

Supplementary Figure 2 	Visual and mathematical walkthrough of the directed 	            viii-ix
Dynamic Time Warping (DTW) analysis. 

Supplementary Figure 3 	Temporal trends in suicidal ideation and COVID-19 mortality              x
during the pandemic period (April 2020–February 2022).

Supplementary Figure 4 	Dynamic alignment of depressive symptoms with SI across 	    xi
sex and age groups    	    

Supplementary Figure 5 	Dynamic alignment of anxiety symptoms with SI across 		   xii
sex and age groups   	     

Supplementary Figure 6 	Dynamic alignment of loneliness symptoms with SI 		  xiii
across sex and age groups.          

Supplementary Figure 7 	Dynamic alignment of worry symptoms with SI across 		  xiv
sex and age groups.  	   

Supplementary Figure 8 	Dynamic alignment of COVID-19 specific stressors with 		   xv
SI across sex and age groups	

Supplementary Figure 9		Dynamic alignment across baseline depression severity 	  	  xvi  		(IDS < 26 vs IDS ≥ 26)

Supplementary Figure 10	Dynamic alignment across baseline anxiety severity                   	 xvii		(BAI < 33 vs BAI ≥ 33)

Supplementary Figure 11	Dynamic alignment across suicidal ideation variability		xviii
subgroups (low vs high variance)	  	

Supplementary Figure 12	Undirected symptom network across all domains 		  xix
(NESDA participants only)             

Supplementary Figure 13	Undirected dynamic effects of all symptoms relative to 	                 xx
suicidal ideation (NESDA participants only)	  	 

Supplementary Figure 14	Directed dynamic effects of all symptoms relative to suicidal 	  xxi
ideation (NESDA participants only)	    	

			

**Supplementary Figure 1**: Flow chart of inclusion/exclusion process.

**Supplementary Table 1.** Baseline characteristics of included vs. excluded participants from the NESDA/NESDO/NOCDA cohorts.

|  | Current study |  | P** |
| --- | --- | --- | --- |
|  | Included (n=307) | Excluded * (n=818) |  |
| **Socio-demographics** |  |  |  |
| Age (SD) | 44.8 (12.1) | 44.6 (13.2) | .735 |
| Female sex (%) | 189 (61.6%) | 535 (65.4%) | .130 |
| High level of education (%) | 118 (38.4%) | 372 (45.5%) | .063 |
| **Comorbidity** |  |  |  |
| Lifetime disorder (%) | 307 (100%) | 552 (67.5%) | <.001 |
| Number of lifetime disorders (SD) | 3.38 (1.38) | 1.76 (1.67) | <.001 |
| Chronicity (SD) | 2.33 (0.77) | 1.24 (1.08) | <.001 |
| **Vaccination attitude** |  |  |  |
| Willing to vaccinate | 220 (87.0%) | 585 (87.7%) | .825 |
| **Functional scores** |  |  |  |
| Avg QIDS pre COV (IQR) | 8.8 (5.8-11.5) | 3.3 (2.0-6.0) | <.001 |
| Avg BAI pre COV (IQR) | 11.6 (6.1-17.1) | 4.0 (1.6-8.0) | <.001 |
| Avg PSWQ pre COV (IQR) | 33.8 (26.3-41.4) | 20.8 (15.0-29.2) | .284 |
| Avg JGLS pre COV (IQR) | 3.0 (1.0-5.0) | 1 (0.0-2.5) | <.001 |
| Avg QIDS during COV (IQR) | 8.7 (6.0-13.0) | 3.0 (2.0-5.0) | <.001 |
| Avg BAI during COV (IQR) | 11.7 (5.4-19.7) | 2.3 (0.0-6.4) | <.001 |
| Avg PWQR during COV (IQR) | 35.0 (27.7-43.3) | 21.0 (13.7-28.3) | .157 |
| Avg JGLS during COV (IQR) | 4.0 (2.3-5.6) | 1.3 (0.7-2.7) | <.001 |

*Participants without a lifetime psychiatric diagnosis and those without any change in suicidal ideation (IDS item 12) during the pandemic were excluded from the analytical sample. **p was estimated by the Chi square test for categorical variables, t-tests for normally distributed variables and the Kruskal-Wallis test for non-normally distributed variables. Comorbidity is assessed through CIDI (Composite International Diagnostic Interview*)* diagnostic interviews based on DSM-VI criteria. IQR denotes Interquartile range (25th and 75th percentiles); QIDS denotes quick inventory of depressive symptomatology; BAI denotes Beck Anxiety Inventory; PWQR denotes Penn State Worry Questionnaire; JGLS denotes de Jong-Gierveld Loneliness Scale. Range: QIDS (0-27), BAI (0-63), PWQR (16-90), JGLS (0-6). 

**Sample R script** ([https://osf.io/gbaw2/](https://osf.io/gbaw2/))

Note on Script and Methodology: > This sample script accompanied an earlier DTW paper we published. Although it reflects different outcomes, it utilizes the same mathematical principle of non-linear temporal alignment applied in this study. For a comprehensive walkthrough of the DTW methodology, parameter selection (such as the Sakoe–Chiba band), and clinical interpretation, we refer to the following tutorial:

Kopland MCG, Giltay EJ. Dynamic Time Warp (DTW) as a scalable, data-efficient, and clinically relevant analysis of dynamic processes in patients with psychiatric disorders: a tutorial. J Eat Disord. 2025;13(1):230.

**COVID-19-specific exposures and responses (English Translation)**

Item (response options: 1=totally disagree – 5=totally agree)

Perceived Impact (mental burden – 9 items)

- Because of this period the quality of my sleep is worse

- This period makes me consume more snacks and sweets

- This period makes me drink more alcohol

- This period makes me more emotional

- In this period I’m having more nightmares

- In this period it’s hard to concentrate

- In this period I’m more often lonely

- This period makes me sad

- In this period I’m more often irritable and tense

Fear of Infection – 6 items

- I fear to become infected with corona

- Because of the threat of the virus I don’t leave my home anymore

- This period makes me fearful

- I intensively follow the news about the virus through TV newspaper and or social media

- Because of the threat of the virus I am anxious of getting close to other people

- I strictly follow the rules to prevent contamination and spread of the virus

Positive Coping – 5 items

- In this period I feel more connected to society

- It is no problem to enjoy myself while being at home more often

- I have confidence that the Netherlands will overcome this crisis

- Despite the virus I stay active (household tasks, gardening, walking, sporting, yoga)

- Despite the virus I actively maintain (via phone or online) contacts with friends

**Supplementary Figure 2.** Visual and mathematical walkthrough of the directed Dynamic Time Warping (DTW) analysis.
Panels A and B illustrate the symptom trajectories of suicidal ideation and sad mood across 13 assessment points. The black dotted lines show how individual observations from one symptom trajectory are matched to observations from the other trajectory using DTW. A time window of 1 was applied, meaning that each alignment step could shift by no more than one next assessment point. Panel A shows the alignment when suicidal ideation is evaluated as preceding sad mood, whereas Panel B shows the reverse ordering, with sad mood evaluated as preceding suicidal ideation. Panels C and D show the corresponding cost matrices under the "symmetric2" time-window constraint. The highlighted cells represent the optimal warping path, that is, the sequence of matches that minimizes the total discrepancy between both symptom trajectories. The alignment starts in the lower-left corner and proceeds toward the upper-right corner. Red cells indicate the selected path cells, whereas orange cells indicate off-path cells that were considered but not included in the final optimal alignment. The final accumulated DTW distance was 18 when suicidal ideation was aligned before sad mood and 8 when sad mood was aligned before suicidal ideation. Panel E shows how these two DTW distances were converted into two relative directed distance statistics. For suicidal ideation preceding sad mood, the directed distance was -0.28. For sad mood preceding suicidal ideation, the directed distance was 0.28. Because the smaller raw DTW distance was obtained when sad mood was aligned before suicidal ideation, the results suggest that changes in sad mood temporally preceded changes in suicidal ideation in this example. Panel F displays the “symmetric2” step pattern, which defines the allowed local matching steps between the query and reference time series during the DTW alignment. For further explanation see Kopland MCG, Giltay EJ. Dynamic Time Warp (DTW) as a scalable, data-efficient, and clinically relevant analysis of dynamic processes in patients with psychiatric disorders: a tutorial. J Eat Disord. 2025;13(1):230.

**Supplementary Figure 3.** Temporal trends in suicidal ideation and COVID-19 mortality during the pandemic period (April 2020–February 2022).
Top panel: Mean suicidal ideation (SI) scores (± SE) over time in the analytical sample of participants included in the DTW analyses (n = 307).
Bottom panel: Weekly COVID-19 mortality in the Netherlands, annotated with major national public health measures (e.g., lockdowns, curfews, and reopening’s). Note that this figure includes all respondents regardless of inclusion in the primary DTW analysis, and thus reflects broader population-level trends.

**Supplementary Figure 4.** Dynamic alignment of depressive symptoms with SI across sex and age groups.
Undirected DTW analysis was used to compute the temporal distance between depressive symptoms (IDS items) and SI. Group effects by sex (male, female) and age (<65 years, ≥65 years) are indicated. Smaller distances indicate greater co-fluctuation over time. P-values for the item compared with SI are displayed to the right of each item, with p < 0.05 considered statistically significant. Group effects are provided and significance is indicated with a letter on the right of the overall p value of the item. Items low energy (IDS5), low self-esteem (IDS16), and sad mood (IDS13) showed the closest temporal association with SI across groups (p<0.001). In contrast, somatic symptoms such as hypersomnia (IDS4), *mid-nocturnal insomnia* (IDS2) sleep onset insomnia (IDS1), and increased appetite (IDS7) were least aligned with SI. Significant group differences were observed for Increased appetite (IDS7), Increased weight (IDS9) and *early morning insomnia* (IDS3). 

**Supplementary Figure 5.** Dynamic alignment of anxiety symptoms with SI across sex and age groups.
Undirected DTW analysis was used to compute the temporal distance between anxiety symptoms (BAI items) and SI. Smaller distances indicate greater co-fluctuation over time. P-values for the item compared with SI are displayed to the right of each item, with p < 0.05 considered statistically significant. Group effects are provided and significance is indicated with a letter on the right of the overall p value of the item. The cognitive-affective symptom fear of losing control (BAI14, p = 0.005) and faint/light-headedness (BAI19, p = 0.007) were significantly aligned with SI. A significant group effect was found for scared (BAI17), reflecting differences between adult males (65-) and adult females (65-). No other significant subgroup differences were found.

**Supplementary Figure 6.** Dynamic alignment of loneliness symptoms with SI across sex and age groups.
Dynamic time warping was applied to loneliness items from the De Jong Gierveld Loneliness Scale to examine their alignment with SI. Smaller distances indicate greater co-fluctuation over time. P-values for the item compared with SI are displayed to the right of each item, with p < 0.05 considered statistically significant. Group effects are provided and significance is indicated with a letter on the right of the overall p value of the item. Emotional loneliness item feeling abandoned was significantly aligned with SI and feeling lonely was moderately aligned with SI p = 0.051, but no significant group differences were observed (all p > 0.05). Missing social contacts demonstrated the least similarity from SI.

**Supplementary Figure 7.** Dynamic alignment of worry symptoms with SI across sex and age groups.
Undirected dynamic time warping (DTW) analysis was used to compute the temporal distance between individual items of the Penn State Worry Questionnaire (PSWQ) and SI. Smaller distances indicate greater co-fluctuation over time. P-values for the item compared with SI are displayed to the right of each item, with p < 0.05 considered statistically significant. Group effects are provided and significance is indicated with a letter on the right of the overall p value of the item. Among all items, only "overwhelming worries" (W1) demonstrated a significant association with SI (p = 0.004), with smaller distances indicating closer temporal alignment. Group effects were observed for "no control over worrying" (W3; p = 0.049) and "overwhelming worries" (W1; p = 0.004), showing greater alignment with SI in younger men (<60 years) compared to women.

**Supplementary Figure 8.** Dynamic alignment of COVID-19 specific stressors with SI across sex and age groups.
Undirected DTW analysis was used to compute the temporal distance between COVID-19-related items and SI. Smaller distances indicate stronger temporal alignment. P-values indicate group-level similarity between each item and SI, with p < 0.05 considered statistically significant. Group differences by age and sex are shown on the right and are denoted with lettered superscripts (see legend below). No COVID-19 items were significantly aligned with SI (all p > 0.05), suggesting low temporal association overall. Significant group effects were found for four items: fearing COVID-19 (Item 1, group f), strictly following COVID rules (Item 21, p < 0.001, group d), intensively following the news (Item 8, p < 0.001, group e), and anxiety about close contacts (Item 20, p < 0.001, group e).

**Supplementary Figure 9.** Dynamic alignment across baseline depression severity (IDS<26 vs IDS≥26)
This figure summarizes the dynamic time warp (DTW) distances between individual symptoms and suicidal ideation (IDS12) across six domains: COVID-related stressors, anxiety (BAI), depression (IDS), loneliness (DJGLS), worry (PSWQ), and happiness. The zero point represents the average distance across all symptoms, such that negative values indicate stronger temporal alignment with SI and positive values indicate weaker alignment. Items showing significantly smaller distances (p < .05) are interpreted as meaningfully co-varying with SI over time. Error bars represent 95% confidence intervals around the estimated distances. Group effect statistics are shown as F-values from ANOVA tests, with corresponding p-values.

**Supplementary Figure 10.** Dynamic alignment across baseline depression severity (BAI<33 vs BAI≥33)
This figure summarizes the dynamic time warp (DTW) distances between individual symptoms and suicidal ideation (IDS12) across six domains: COVID-related stressors, anxiety (BAI), depression (IDS), loneliness (DJGLS), worry (PSWQ), and happiness. The zero point represents the average distance across all symptoms, such that negative values indicate stronger temporal alignment with SI and positive values indicate weaker alignment. Items showing significantly smaller distances (p < .05) are interpreted as meaningfully co-varying with SI over time. Error bars represent 95% confidence intervals around the estimated distances. Group effect statistics are shown as F-values from ANOVA tests, with corresponding p-values.

**Supplementary Figure 11. Dynamic alignment across suicidal ideation variability subgroups (low vs. high variance).** 
This figure displays the dynamic time warp (DTW) distances between individual symptoms and suicidal ideation (SI) across six domains, stratified by low (n = 155) and high (n = 152) within-person standard deviation of the SI item (IDS12). The zero-baseline represents the empirical mean distance across all symptom pairs; negative values indicate stronger temporal alignment (smaller distance/co-variation) with SI, whereas positive values indicate weaker alignment. Point estimates are displayed with their corresponding 95% confidence intervals and unadjusted two-tailed p-values.

**Supplementary Figure 12.** Undirected symptom network across all domains (in 241 NESDA participants only).
QIDS denotes quick inventory of depressive symptomatology; BAI denotes Beck Anxiety Inventory; PWQR denotes Penn State Worry Questionnaire; JGLS denotes de Jong-Gierveld Loneliness Scale. Nodes represent individual items. Edges reflect significant partial correlations between items, with edge thickness proportional to strength. Node colour separates symptom domains. The network is undirected and based on pooled data across all 307 participants. Red edges highlight the temporal alignments between suicidal ideation (IDS12) and co-fluctuating symptoms for visual emphasis

**Supplementary Figure 13.** Undirected dynamic effects of all symptoms relative to suicidal ideation (in 241 NESDA participants only).
This figure summarizes the dynamic time warp (DTW) distances between individual symptoms and suicidal ideation (IDS12) across six domains: COVID-related stressors, anxiety (BAI), depression (IDS), loneliness (DJGLS), worry (PSWQ), and happiness. The zero point represents the average distance across all symptoms, such that negative values indicate relative stronger temporal alignment with SI and positive values indicate relative weaker alignment. Only items showing significantly smaller distances (p < .05) are interpreted as meaningfully co-varying with SI over time. Error bars represent 95% confidence intervals around the estimated distances. Group effect statistics are shown as F-values from ANOVA tests, with corresponding p-values.

**Supplementary Figure 14.** Directed dynamic effects of all symptoms relative to suicidal ideation (in 241 NESDA participants only).
This figure displays the dynamic time warp (DTW) distances for all symptoms in relation to suicidal ideation (IDS12). Positive values indicate a leading effect where changes in the symptom tend to precede changes in SI, whereas negative values indicate a lagging effect where the symptom tends to follow fluctuations in SI. Items  are grouped by domain: anxiety (BAI), depression (IDS), loneliness (DJGLS), worry (PSWQ), happiness, and COVID-19 stress- and behaviour related symptoms. Error bars represent 95% confidence intervals around the estimated effects. Items with p < .05 are considered to show significant directed temporal associations with SI.
